# Histones Induce the Procoagulant Phenotype of Endothelial Cells through Tissue Factor Up-Regulation and Thrombomodulin Down-Regulation

**DOI:** 10.1371/journal.pone.0156763

**Published:** 2016-06-03

**Authors:** Ji Eun Kim, Hyun Ju Yoo, Ja Yoon Gu, Hyun Kyung Kim

**Affiliations:** 1 Department of Hemato-oncology, Healthcare Innovation Park, Seoul National University Bundang Hospital, Gyeonggi-do, South Korea; 2 Department of Laboratory Medicine and Cancer Research Institute, Seoul National University College of Medicine, Seoul, Korea; University of Sheffield, UNITED KINGDOM

## Abstract

The high circulating levels of histones found in various thrombotic diseases may compromise the anticoagulant barrier of endothelial cells. We determined how histones affect endothelial procoagulant tissue factor (TF) and anticoagulant thrombomodulin (TM). Surface antigens, soluble forms, and mRNA levels of TF and TM were measured by flow cytometry, ELISA, and real-time RT-PCR, respectively. TF and TM activity were measured using procoagulant activity, thrombin generation, or chromogenic assays. Involvement of the toll-like receptor (TLR) was assessed using the neutralizing antibodies. Histones dose-dependently induced surface antigens, activity and mRNA levels of endothelial TF. Histone-treated endothelial cells significantly shortened the lag time and enhanced the endogenous thrombin potential of normal plasma, which was normalized by a TF neutralizing antibody. Histones induced phosphatidylserine and protein-disulfide isomerase expression in endothelial cells. Histones also reduced the surface antigen, activity, and mRNA levels of endothelial TM. Polysialic acid and heparin reversed the histone-induced TF up-regulation and TM down-regulation. Activated protein C did not affect the TF up-regulation, but interrupted TM down-regulation. TLR2, and TLR4 inhibitors partially blocked the TF up-regulation. Histones induced the endothelial procoagulant phenotype through TF up-regulation and TM down-regulation. The effects of histones were partly mediated by TLR2, TLR4. Strategies to inhibit the harmful effects of histones in endothelial cells may be required in order to prevent a thrombotic environment.

## Introduction

Histones exist predominantly in a form of nucleosome associated with DNA within the cell nucleus. In a phenomenon referred to as extracellular traps, histones can be released into the blood circulation by inflammatory stimuli from peripheral leukocytes, such as neutrophils, mast cells, eosinophils, and monocytes [1]. The extracellular traps are actively formed in patients with inflammatory, autoimmune, and thrombotic diseases [2–5]. Therefore, the contribution of histones to the process of coagulation has received recent attention [6]. Histones induce platelet activation [7, 8] and enhance plasma thrombin formation *via* binding to protein C and thrombomodulin (TM) [9].

Endothelial cells demonstrate constitutive anticoagulant properties that serve to suppress coagulation activation. These anticoagulant properties are mediated by TM. When thrombin binds to TM on the endothelial surface, it activates protein C, which can extinguish coagulation amplification through inactivation of factors V and VIII [10].

Tissue factor (TF) initiates coagulation by binding coagulation factor VII [11]. TF is constitutively expressed by perivascular cells, such as pericytes and fibroblasts, whereas it is not expressed by resting endothelial cells. In certain pathologic environments, TF can be induced in endothelial cells and contribute to local fibrin formation [12]. TF is normally encrypted on the cell surface, but can be fully activated (decrypted) by certain stimuli [13]. Phosphatidylserine, which normally exists on the inner layer of the plasma membrane, could enhance the procoagulant activity of TF by transferring to the outer layer [14]. In addition, the results from recent reports have suggested that TF activity was regulated by the formation of a disulfide bond within the extracellular domain of TF through protein-disulfide isomerase (PDI) [15].

As high levels of circulating histones are associated with various inflammatory and thrombotic diseases [2–4, 16, 17], it is plausible that circulating histone levels may compromise the anticoagulant barrier of endothelial cells. To date, there have been no reports regarding on the effects of histones on the endothelial anticoagulant phenotype. This study investigated how histones affected procoagulant TF and anticoagulant TM expression in endothelial cells. Additionally, the involvement of the toll-like receptor (TLR) in mediating the effects of histones was examined.

## Materials and Methods

### Cell culture

The human endothelial cell line, EA.hy926, was purchased from ATCC. EA.hy926 was maintained in DMEM medium (WelGENE, Seoul, South Korea) supplemented with 10% fetal bovine serum (Gibco, Grand Island, NY, USA). The cells were starved without serum for one hour prior to stimulation with calf thymus histones (Roche Diagnostics, IN, USA). After four hours, the cells and supernatants were harvested using 0.25% trypsin-EDTA solution (ThermoFisher Scientific, Waltham, MA, USA).

### Flow cytometric analysis

The cells were stained with rabbit anti-TF antibody conjugated with alexa fluor 647 (Bioss Inc., Woburn, MA, USA). Phosphatidylserine was detected with PE-conjugated annexin V (BD Biosciences, Franklin Lakes, NJ, USA). Rabbit IgG conjugated with alexa fluor 647 (Bioss Inc.) was used as an isotype control. DyLight^TM^ 488-conjugated mouse anti-PDI antibody (clone 1D3; Enzo Life Sciences, Farmingdale, NY, USA), PE-conjugated mouse anti-TM antibody (BD Biosciences), and 7AAD (Beckman coulter, Brea, CA, USA) were used. In select experiments, the mouse anti-TM antibody (clone PBS-01; Abcam, Cambridge, UK) against epidermal growth factor (EGF) domain 5 of TM was stained. Subsequently, FITC-conjugated goat anti-mouse IgG (Santa Cruz, Dallas, TX, USA) was used as a second antibody.

Human recombinant histones H1, H2A/H2B, H3.3, and H4 (New England BioLabs Inc., Ipswich, MA, USA) were used. Potential histone inhibitors, polysialic acid (PSA, 62.5 μM; Sigma-Aldrich, St Louis, MO, USA), heparin (100 U/mL, Greencross, South Korea), and activated protein C (APC, 100 nM; Haematologic technologies Inc., River Road, VT, USA) were pre-incubated with histones and then used as stimulants. EA.hy926 cells were pre-incubated with anti-TLR2 antibody (50 μg/mL, eBioscience, San Diego, CA, USA), anti-TLR4 antibody (50 μg/mL, eBioscience) for 1 h before histone stimulation. Mouse IgG_2a,K_ antibody (50 μg/mL, eBioscience) was used as an isotype control. GM6001 (10 μM, Enzo Life Sciences) was used to suppress matrix metalloproteinase (MMP) activity.

### Detection of soluble proteins by ELISA

The soluble TF was measured using a human Coagulation Factor III/TF DuoSet (R&D Systems, Minneapolis, MN, USA) ELISA performed in accordance with the instructions provided by the manufacturer. The soluble TM was measured using a Thrombomodulin Human ELISA Kit (Abcam) according to the manufacturer’s protocol.

### Detection of mRNA expression by quantitative RT-PCR

Total RNA was extracted from EA.hy926 cells using TRIzol (Life Technologies, Gaithersburg, MD, USA) reagent according to instructions of the manufacturer. Synthesis of cDNA was performed with 1 μg total RNA using the GoScript reverse transcription system (Promega, Fitchburg, WI, USA). Real-time PCR amplification was carried out using the ABI Prism 7000 Sequence Detection system (Applied Biosystems, Foster City, CA, USA), TaqMan Universal PCR Master Mix, and TF- and TM-specific primers and 6-carboxyfluorescene-labeled probe sets (Applied Biosystems) for quantitative gene expression. Expression levels of the genes were normalized to internal glyceraldehyde 3-phosphate dehydrogenase primer/probe pair levels (VIC MGB probe, primer limited, Applied Biosystems). These were presented as relative expression levels.

### TF procoagulant activity

After stimulation with histones for 4 h, EA.hy926 cells were harvested. Cells were resuspended to 5 x 10^6^/mL in PBS and 20 μL of cell suspension was added in a cuvette. The plasma (80 μL; Pool Norm; Diagnostica Stago, Asnieres, France) which was anticoagulated with 3.2% sodium citrate was mixed with the cell suspension and the formation of a clot was induced by the addition of 100 μL of pre-warmed 0.025 M CaCl_2_. The clotting time was measured by ST Art (Diagnostica Stago). The standard curve was produced using HemosIL RecombiPlasTin 2G (Instrumentation Laboratory, Bedford, MA, USA).

### Thrombin generation assay (TGA)

Thrombin generation was measured in a Fluoroskan Ascent (ThermoFisher Scientific) as described by Lecompte et al [18]. Briefly, 80 μL plasma (Pool Norm) was mixed with 20 μL of 1 x 10^4^ EA.hy926 cells stimulated with histones for 4 h. Subsequently, 20 μL substrate (FluCa-Kit, Thrombinoscope BV, Maastricht, Netherlands) was added. The thrombin generation was measured using Thrombinoscope software (Thrombinoscope BV). In some experiments, antibodies against human TF (clone VD8, 30 μg/mL; American Diagnostica Inc., Stamford, CT, USA) and recombinant annexin V (10 μg/mL, BD Biosciences), anti-PDI antibody (clone RL90, 10 μg/mL; Abcam), glutathione (7.5 mM, Sigma-Aldrich), and quercetin (200 μM, Sigma-Aldrich) were used as inhibitors.

### TM activity assay

Twenty μL of 1 x 10^4^ EA.hy926 cells was mixed with 80 μL reaction buffer (Tris 0.05M, NaCl 0.25 M, CaCl_2_ 4 mM, pH 8.0), containing 4 μg/mL human protein C (Haematologic technologies Inc.) and 0.03 U/mL human thrombin (Sigma-Aldrich). Subsequently, 20 μL of the substrate, S-2366 (Chromogenix, Milano, Italy) was supplied at a final concentration of 200 μM. The absorbance from 405 nm was measured at 37°C using a Multiscan GO (ThermoFisher Scientific) for 90 minutes. The standard curve was generated with rabbit TM (Haematologic technologies Inc.).

### Statistical analysis

All statistical analyses were performed using SPSS 21.0 for Windows (SPSS Inc., Chicago, IL, USA). All data are presented as mean ± standard error of the mean. The data were combined data from 3 or more different experiments. Data comparisons were conducted using t-tests. *P*-values of < 0.05 were considered to be statistically significant.

## Results

### Histones induce TF expression in endothelial cells

**Fig 1:**
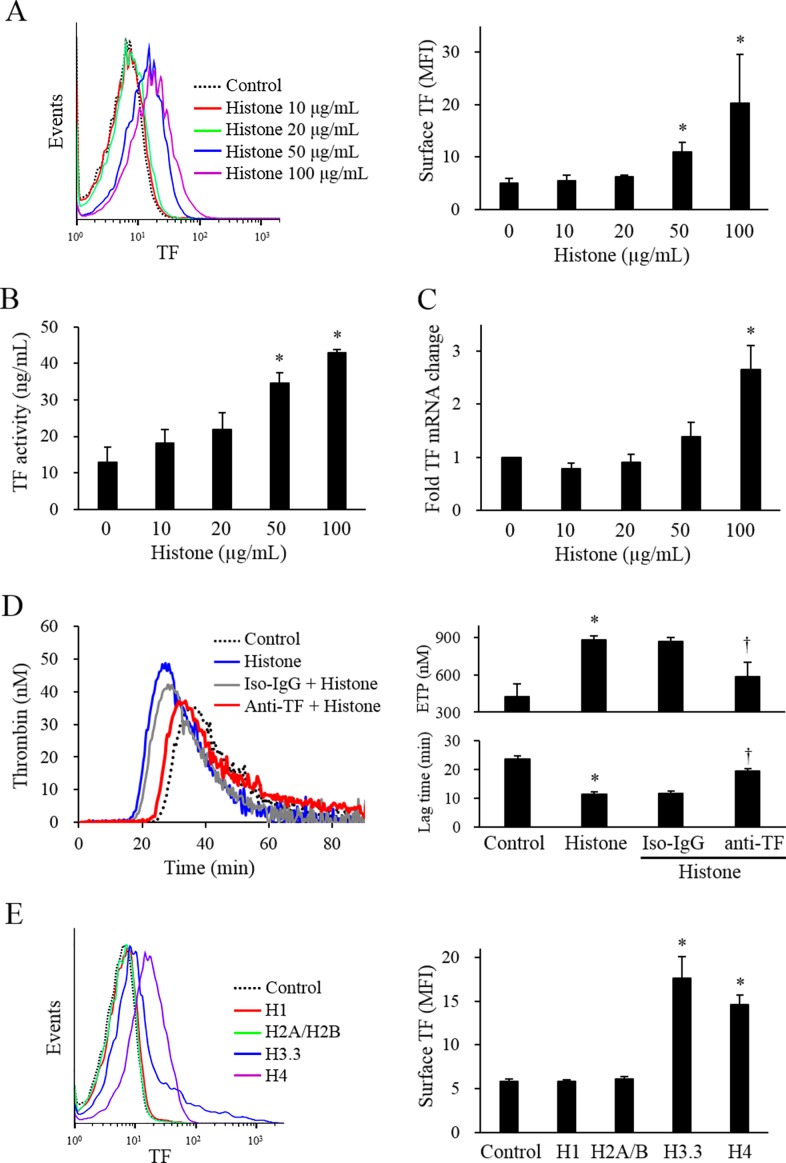
Histones induced TF expression in endothelial cells. (A) After EA.hy926 cells were stimulated with various levels of calf thymus histones for 4 h, the surface expression of TF antigens was determined by flow cytometry. A summary of the TF surface expression stimulated by calf thymus histones is shown in the right panel. (B) The procoagulant activity of TF was measured in EA.hy926 cells incubated with various levels of calf thymus histones for 4 h. (C) The expression of TF mRNA was quantified using real-time RT-PCR in EA.hy926 cells incubated with various levels of calf thymus histones for 3 h. (D) EA.hy926 cells were either stimulated with or without 50 μg/mL calf thymus histones for 4 h, and incubated with PBS (control), mouse isotype IgG (Iso-IgG, 30 μg/mL), or inhibitory TF antibody (anti-TF, 30 μg/mL) for 10 minutes. The ETP and lag time were analyzed using a thrombin generation assay. (E) EA.hy926 cells were stimulated with individual human recombinant histone (20 μg/mL H1, H2A/H2B, H3.3, H4) for 4 h, and the expression of surface TF antigens was determined by flow cytometry. All data are presented as mean ± SEM. The data were combined data from 3 or more different experiments. * *P<*0.05 vs. control (calf thymus histones not treated).

Calf thymus histones dose-dependently induced TF expression in endothelial cells (Fig 1A). Corresponding to the TF antigen expression, the TF activity, as measured by procoagulant activity, was dose-dependently increased (Fig 1B). Calf thymus histones also up-regulated the TF mRNA level in a dose-dependent manner (Fig 1C). The histone-treated endothelial cells significantly shortened the lag time and enhanced the endogenous thrombin potential (ETP), as compared with the endothelial cells without histone treatment. The neutralizing antibody against TF normalized the shortened lag time and enhanced ETP in histone-treated endothelial cells to control levels (Fig 1D). To investigate the relative effect of individual histones on TF expression of endothelial cells, they were treated with each subtype of histones. H3.3 and H4 were more potent than H1 or H2A/2B in accomplishing histone-induced TF up-regulation (Fig 1E). The amounts of soluble TF in the culture supernatants were not increased in histone-treated cells (data not shown).

We further evaluated that histones induced cell damage. Cells were stained with annexin V and 7AAD, and analyzed by flow cytometry. The annexin V-7AAD double positive cells were increased in calf thymus histones-stimulated cells then in control cells (S1 Fig). In the annexin V negative population, TF expression was still induced (S2 Fig).

### Histones increase phosphatidylserine and PDI expression in endothelial cells

**Fig 2:**
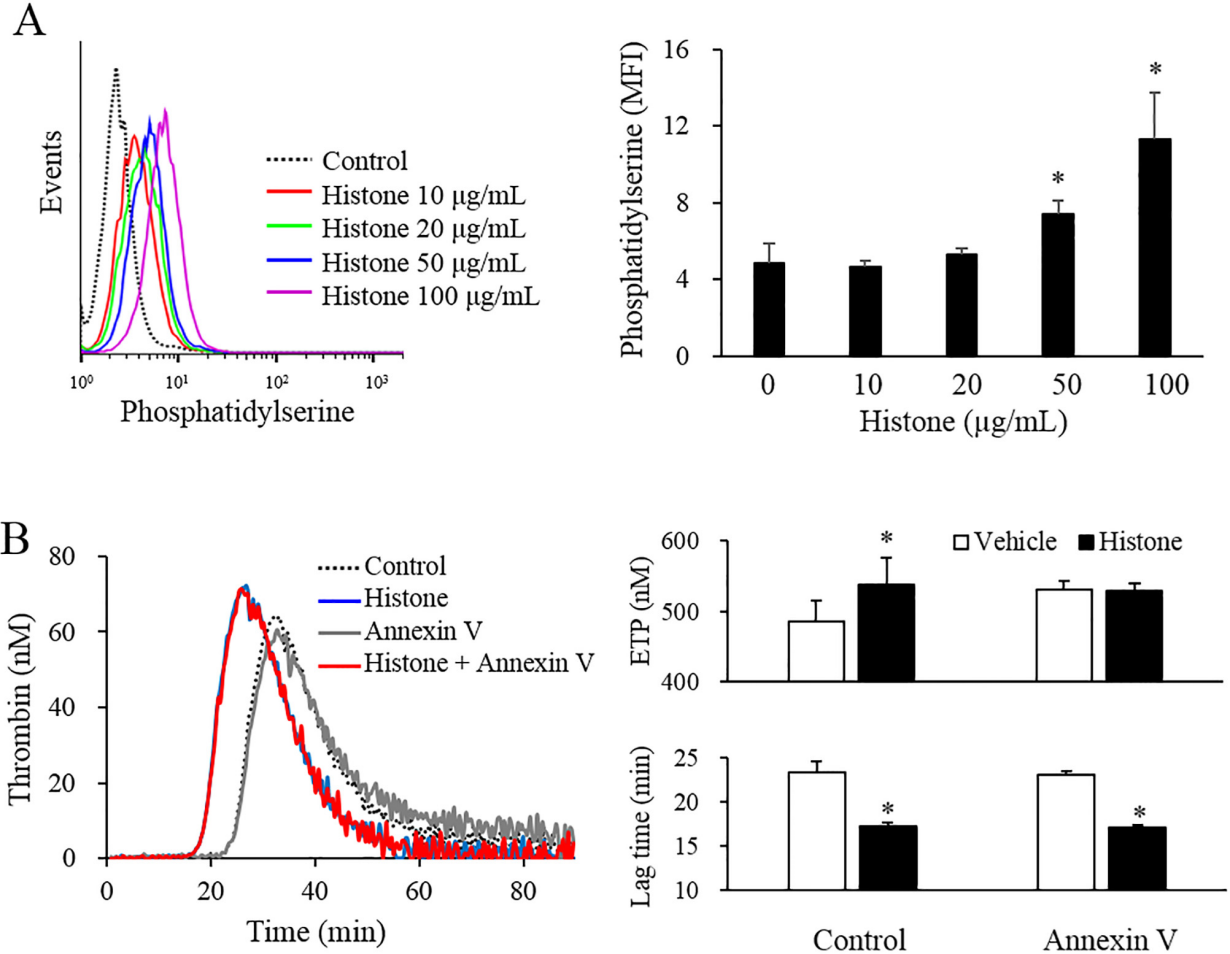
Histones induced phosphatidylserine exposure in endothelial cells. (A) EA.hy926 cells were stimulated with various levels of calf thymus histones for 4 h, and the surface phosphatidylserine level was determined by flow cytometer. (B) EA.hy926 cells were stimulated with or without 50 μg/mL calf thymus histones for 4 h, and incubated with PBS (vehicle) or 10 μg/mL annexin V for 15 min. The ETP and lag time were analyzed using a thrombin generation assay. All data are presented as mean ± SEM. The data were combined data from 3 or more different experiments. * *P<*0.05 vs. control (calf thymus histones not treated).

Calf thymus histones increased surface phosphatidylserine in a dose-dependent manner (Fig 2A). Annexin V was used to cover the exposed phosphatidylserine. The addition of annexin V to histone-treated cells did not modify the ETP or lag time in TGA (Fig 2B). TF activity, as measured by procoagulant activity, was similarly unaffected by the addition of annexin V (S3 Fig)

**Fig 3:**
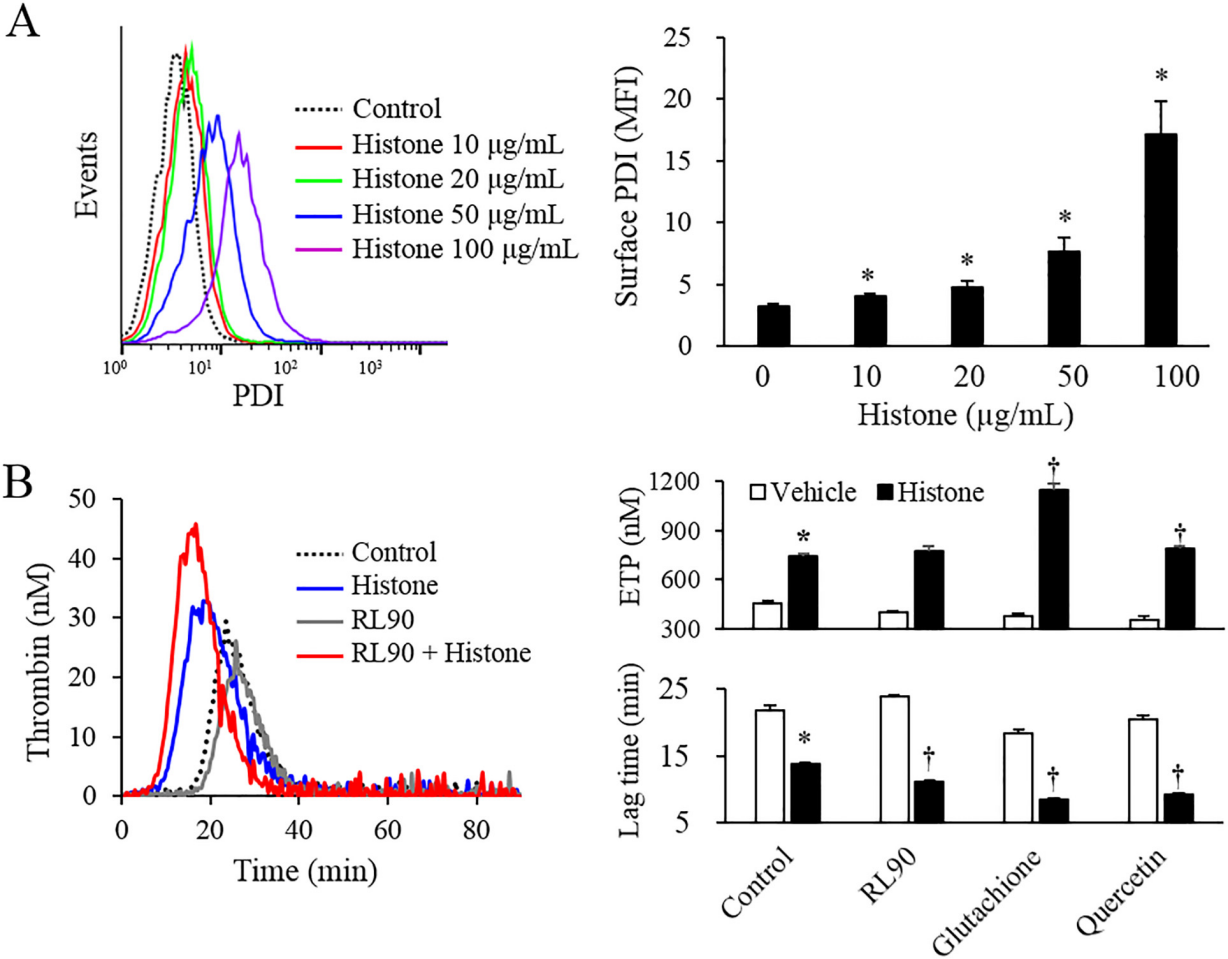
Histones increased PDI expression in endothelial cells. (A) EA.hy926 cells were stimulated with various levels of calf thymus histones for 4 h, and the levels of surface PDI expression were determined by flow cytometry. (B) EA.hy926 cells were pre-incubated with PBS (control) or inhibitors, such as anti-PDI antibody (RL90, 10 μg/mL), glutathione (7.5 mM), and quercetin (200 μM) for 1 h. The cells were then stimulated with or without 50 μg/mL calf thymus histones for 4 h. The ETP and lag time were analyzed using a thrombin generation assay. All data are presented as mean ± SEM. The data were combined data from 3 or more different experiments. * *P<*0.05 vs. control (calf thymus histones not treated), ^†^ *P<*0.05 vs. histone-treated cells.

Calf thymus histones increased the surface PDI expression in a dose-dependent manner (Fig 3A). Several PDI inhibitors were used to block the regulation of TF decryption by PDI. PDI neutralizing antibody (RL90) shortened the lag time in histone-treated cells. Both glutathione and quercetin significantly shortened the lag time and increased the ETP in histone-treated cells (Fig 3B). TF activity, as measured by procoagulant activity, was also increased by the three PDI inhibitors (S4 Fig)

### Histones reduce TM expression in endothelial cells

**Fig 4:**
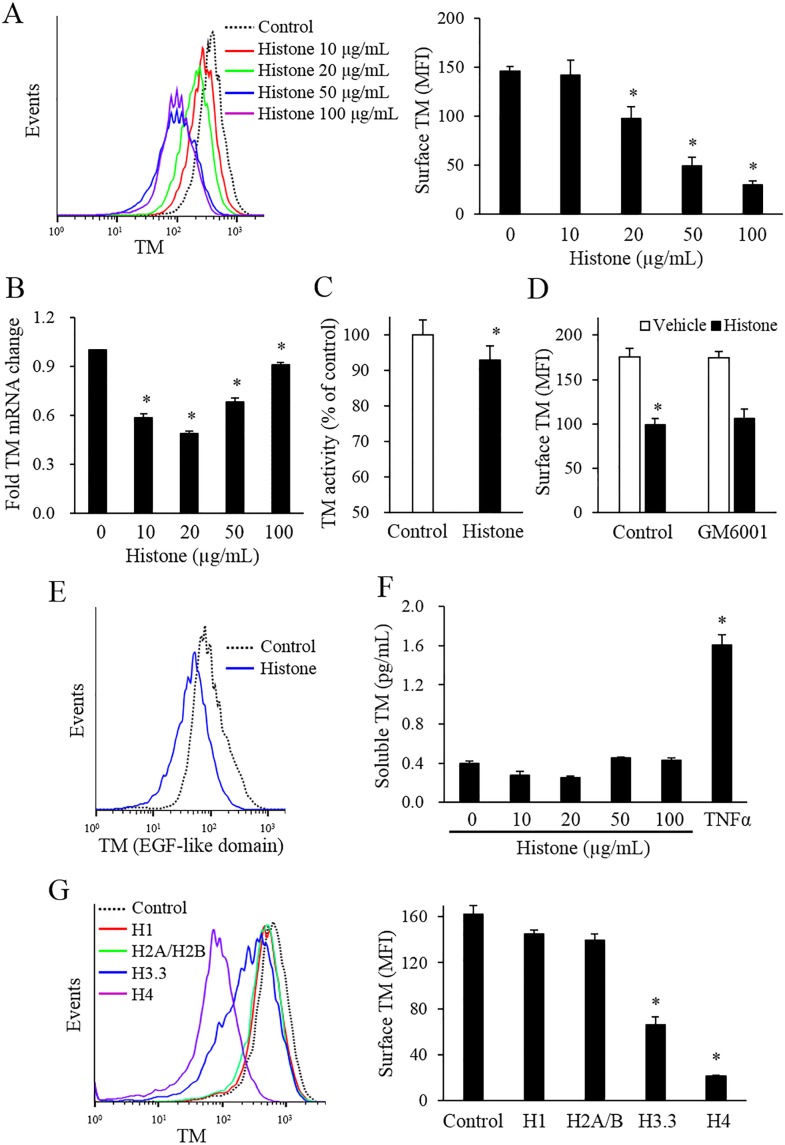
Histones reduced the endothelial surface expression of TM. (A) EA.hy926 cells were treated with various levels of calf thymus histones for 4 h, and the expression levels of surface TM was determined by flow cytometry with an anti-TM antibody (epitope: lectin-like domain). (B) TM mRNA expression levels were quantified by real-time RT-PCR in EA.hy926 cells incubated with various levels of calf thymus histones for 3 h. (C) EA.hy926 cells were treated with 50 μg/mL calf thymus histones for 4 h, and TM activity was measured using the chromogenic assay described in the Materials and Methods section. (D) EA.hy926 cells were pre-incubated with an MMP inhibitor (10 μM GM6001) for 30 min, and then stimulated with or without 50 μg/mL calf thymus histones, for 4 h. The level of surface TM was determined by flow cytometry with the aforementioned anti-TM antibody. (E) EA.hy926 cells were treated with or without 50 μg/mL calf thymus histones for 4 h and the level of surface TM expression was measured using the anti-EGF-like domain of the TM. (F) EA.hy926 cells were treated with various levels of calf thymus histones or 2 ng/mL TNF-α (as a positive control) for 4 h, and the soluble TM levels in the culture supernatants were measured by ELISA. (G) EA.hy926 cells were stimulated with individual human recombinant histones (20 μg/mL H1, H2, H3.3, or H4) for 4 h, and the level of surface expression of TF antigens was determined by flow cytometry. All data are presented as mean ± SEM. The data were combined data from 3 or more different experiments. The flow cytometry results about TM EGF-like domain are representative of 3 experiments. * *P<*0.05 vs. control (calf thymus histones not treated).

Calf thymus histones reduced the expression of endothelial TM in a dose-dependent manner (Fig 4A). TM expression was still reduced by histone stimulation in annexin V negative cell population (S2 Fig). The TM mRNA expression was markedly decreased in low concentrations of histone-treated cells, and remained significantly decreased in high concentrations of histone-treated cells (Fig 4B). TM activity was significantly decreased in histone-treated cells as well (Fig 4C). Because some stimuli can induce the lectin-like domain shedding of surface TM by MMPs [19], we investigated whether an MMP inhibitor could block the histone-induced TM reduction. The MMP inhibitor, GM6001 did not modify the histone-induced TM reduction (Fig 4D). Likewise, the histone-induced TM reduction was still observable through the use of a TM antibody against the EGF domain 5 of TM (Fig 4E). The soluble TM levels were not increased in the culture supernatants of histone-treated cells (Fig 4F). Among the individual histones, H4 was most potent than other subtype of histones in endothelial TM reduction (Fig 4G).

Similar to results obtained from EA.hy926 cells, the primary HUVEC also showed a procoagulant phenotype induced by calf thymus histones (S5 Fig)

#### PSA and heparin neutralize the histone effect in the endothelial phenotype

**Fig 5:**
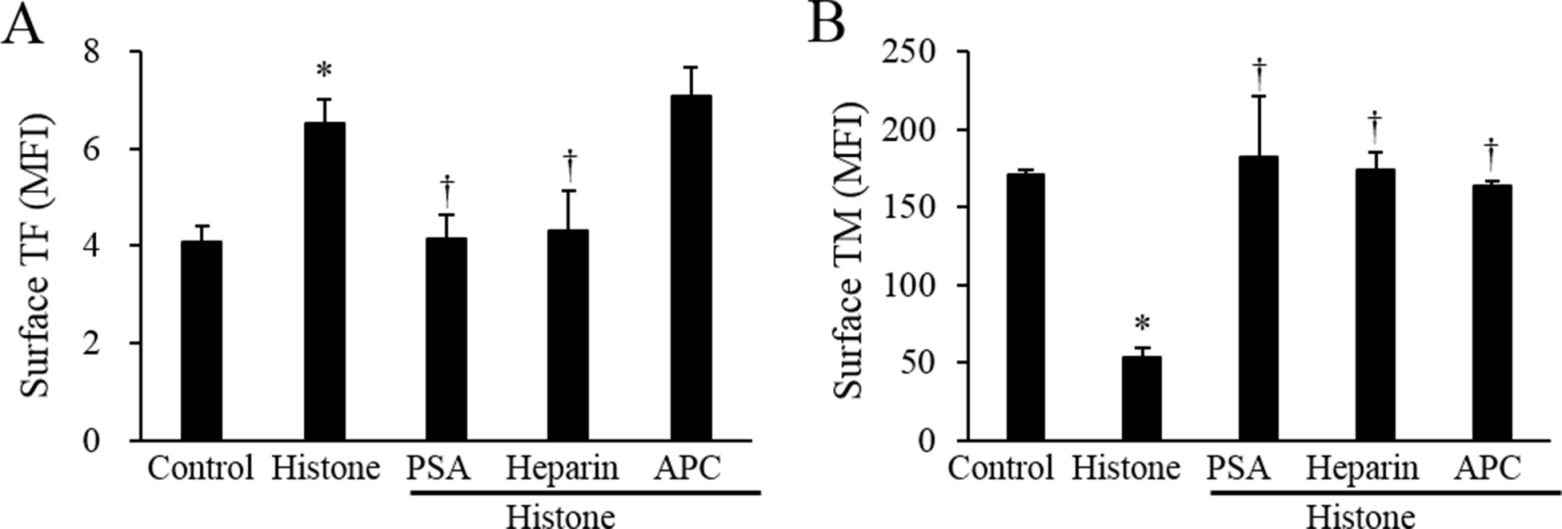
Neutralization of the histone effect by PSA, heparin, and APC. Calf thymus histones were pretreated with or without 62.5 μM PSA, 100 IU/mL heparin, and 100 nM APC for 15 min. The pretreated calf thymus histones (50 μg/mL) were added to the EA.hy926 cells for 4 h. The surface expression levels of TF (A) and TM (B) were measured by flow cytometry. All data are presented as mean ± SEM. The data were combined data from 3 or more different experiments. * *P<*0.05 vs. control (calf thymus histones not treated), ^†^ *P<*0.05 vs. histone-treated.

PSA and heparin are known to bind to histones due to their negatively charged properties [20, 21]. We assessed whether these molecules could neutralize the histone effect on the endothelial procoagulant phenotype. Pretreatment of PSA or heparin in the histone reagent significantly blocked the histone-induced TF up-regulation and TM down-regulation. APC, which can cleave histones [22], could not inhibit the histone-induced TF up-regulation, but completely abolished the histone-induced TM down-regulation (Fig 5). The histone cleavage by APC was shown in S6 Fig.

#### Inhibition of TLR2, TLR4 suppresses the histone effect on the endothelial phenotype

**Fig 6:**
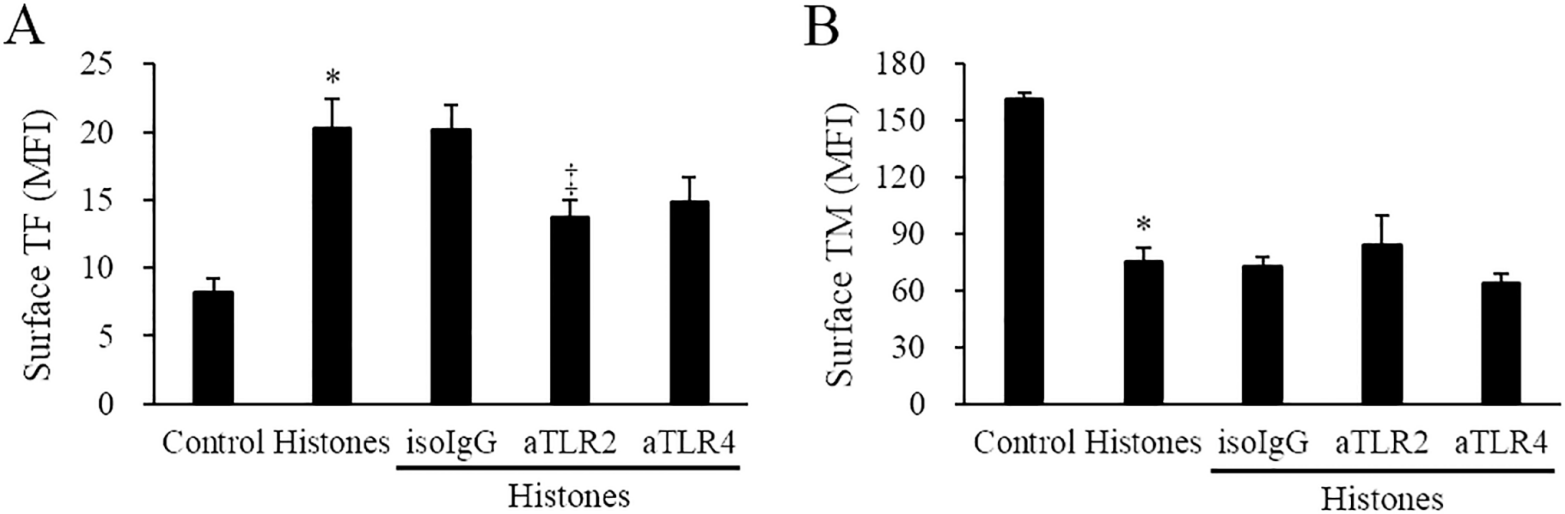
Inhibition of TLR in the histone-induced procoagulant phenotype. EA.hy926 cells were pre-incubated with mouse isotype IgG (Iso-IgG, 50 μg/mL), mouse anti-human TLR2 (aTLR2, 50 μg/mL), or anti-human TLR4 (aTLR4, 50 μg/mL) for 30 min, and then stimulated with or without 50 μg/mL calf thymus histones for 4 h. The surface expression levels of TF and TM were determined by flow cytometry. All data are presented as mean ± SEM. The data were combined data from 3 or more different experiments. * *P<*0.05 vs. control (calf thymus histones not treated), ^†^ *P<*0.05 vs. histone-treated, ^‡^ *P<*0.1 vs. histone-treated.

The neutralizing antibodies against potential histone receptors (TLR2 or TLR4) were pre-incubated with the cells prior to histone stimulation. Anti-TLR2 and partially anti-TLR4 blocked the histone-induced TF up-regulation (aTLR2; *p* = 0.042, aTLR4; *p* = 0.106). Anti-TLR2 and anti-TLR4 did not inhibit the histone-induced TM down-regulation (Fig 6).

## Discussion

Recent reports have described a pathogenic role for histones as prothrombotic substances [6–9, 22]. Histones promote not only platelet activation [7, 8], but also coagulation activation through inhibition of TM-dependent protein C activation [9]. Since histones are cationic proteins, they can bind to anionic TM and inhibit the anticoagulant protein C system. The report from Kowalska et al. has been demonstrated that histones induced the generation of APC in mouse models [23] In this study, we demonstrated a novel role for histones in inducing the endothelial procoagulant phenotype. Histones induced the procoagulant phenotype of endothelial cells by up-regulating the procoagulant TF and down-regulating the anticoagulant TM.

The resting endothelium does not express TF, which is an important initiator of the extrinsic coagulation pathway. However, pathologic stimuli, such as interleukin-6 and endotoxin, can transform the anticoagulant endothelium to be procoagulant. In our results, histones enhanced levels of both TF antigen and activity through TF mRNA synthesis in endothelial cells. In our *in vitro* cell-based TGA, histone-pretreated endothelial cells increased the thrombin generation that was inhibited by the TF neutralizing antibody. Because intravascular TF contributes to the activation of coagulation in various disorders, such as infection, trauma, and malignancy[24], histone-induced endothelial TF expression can be considered to play an essential role in coagulation activation, especially in those clinical conditions associated with high circulating histone levels [4, 16, 25, 26].

TF activity is also affected by phosphatidylserine exposure. Our results showed that histones dose-dependently increased phosphatidylserine exposure in endothelial cells. This finding may be related to the cytotoxic effects of histones. Compared with the paper published by Hu et al [22], the histone-induced cytotoxic intensity was less in our experiment. The difference of cytotoxicity intensity may result from culture conditions. To investigate whether the histone-induced phosphatidylserine externalization contributed to TF activity, annexin V was added to the histone-treated endothelial cells, and the TF activity was then measured. Even after the cover of exposed phosphatidylserine in the histone-treated cells with annexin V, the lag time was not changed. Similar to our results, other studies have demonstrated that TF activity was only partially blocked or not blocked at all by annexin V, suggesting additional molecular mechanisms of TF activation [27–29].

PDI is another known modulator of TF decryption that acts by regulating the redox state of the disulfide bond in the extracellular domain of TF [30]. Endothelial cells express PDI mainly in the endoplasmic reticulum, but also partially on the surface. Interestingly, our results showed that histones dose-dependently increased PDI expression on the surface of endothelial cells. PDI inhibitors were used to investigate the involvement of PDI in TF activity. The PDI inhibitors enhanced the TF activity in histone-treated endothelial cells. Although there have been several reports indicating that PDI inhibitors increased TF activity and thrombosis [14, 31], the expression of PDI in endothelial cells has been suggested to be a negative regulator of coagulation [30]. Consistent with the results of the aforementioned report [30], our results demonstrated that PDI inhibition increased TF activity and indicated that endothelial PDI is a negative regulator of TF activity. It is assumed that the histone-induced PDI increment may be a physiological defense mechanism against the histone-induced procoagulant endothelial phenotype.

TM is a transmembrane glycoprotein expressed in endothelial cells [32]. TM is composed of 5 domains, and the EGF-like domain has catalytic site. [19]. Inflammatory stimuli enhance soluble TM shedding from endothelial cells using several proteases including elastase, proteinase, and cathepsin G [33]. Previous experimental results have indicated that these processes were considerably blocked by MMP inhibitors [34]. In our experiments, however, an MMP inhibitor did not change the histone effect, suggesting that histones did not induce the soluble TM shedding from endothelial cells, but rather decreased the presence of surface TM through reduced mRNA synthesis. Inflammatory stimuli have also been shown to suppress endothelial TM expression [32]. Similar to the aforementioned result, our results support the notion that endothelial TM down-regulation may be a causal factor of a hypercoagulability in the clinical conditions associated with high circulating histone levels.

Several reports have demonstrated that histone H3 and H4 were more cytotoxic and enhanced more thrombin generation and platelet activation than histone H1 and H2 [7–9, 22]. Similarly our results showed that histone H3 and H4 were strong inducer of TF up-regulation and TM down-regulation compared to histone H1 and H2. The results suggest that histone H3 and H4 are more important therapeutic targets for sepsis and other inflammatory diseases.

PSA is a highly negatively charged glycan that can bind to histones [20]. The finding that PSA abolished the histone-induced endothelial procoagulant phenotype suggested a potential role for this agent as an anticoagulant. Heparin can also block the histone effect *via* binding to histones. Additionally, APC that degrades histones [22] could not block the histone-induced TF up-regulation, but completely blocked the histone-induced TM down-regulation. This finding suggested that even the histones degraded by APC may act on the downstream signal for TF transcription. Regarding TM down-regulation, however, the degraded histones were considered to have lost these effects. Because APC is one of the therapeutic candidates for dampening the hypercoagulability of DIC [35], our finding that APC could not block the histone-induced endothelial procoagulant phenotype should be considered as an important limitation of APC therapy.

It has been shown that histones bind to TLR2 and TLR4 [36]. We evaluated the possible role of these receptors in the histone-induced endothelial procoagulant phenotype. TLR2 and partially TLR4 were involved in histone-induced TF up-regulation, whereas they were not involved in histone-induced TM down-regulation. The data suggests that additional activation mechanisms may be required for histone-induced TM down-regulation.

## Conclusions

Our study was the first to demonstrate that histones induced the endothelial procoagulant phenotype through TF up-regulation and TM down-regulation. The histone-induced TF up-regulation was mediated by TLR2, TLR4. These finding suggested that histones play a role in damaging the normal physiologic anticoagulant endothelial surface, resulting in a pathologic procoagulant endothelial surface. Because circulating histone levels are increased in various diseases including sepsis, DIC, cancer, and autoimmune disorders that are associated with thrombosis [2–5, 16], our findings of the histone-induced endothelial procoagulant phenotype may help to elucidate the pathogenesis of the hypercoagulable status in those diseases. Strategies to inhibit the harmful effects of histones in endothelial cells may be required in order to prevent a thrombotic environment.

## Supporting Information

S1 Materials and Methods Cell culture, Western blot. (PDF)

S1 Fig Histones induced cell damage. (PDF)

S2 Fig The expression of TF and TM in undamaged cells by histones-stimulation. (PDF)

S3 Fig Annexin V did not affect the induced TF activity of histone-stimulated cells. (PDF)

S4 Fig TF activity of histone-stimulated cells with protein-disulfide isomerase (PDI) inhibitors. (PDF)

S5 Fig Histones influenced the expression of several proteins on HUVECs. (PDF)

S6 Fig Western blot analysis for the degradation of histone by activated protein C (APC). (PDF)
