# Supplementary material for: Histones Induce the Procoagulant Phenotype of Endothelial Cells through Tissue Factor Up-Regulation and Thrombomodulin Down-Regulation
Source: PLoS One. 2016 Jun 3;11(6):e0156763. doi: 10.1371/journal.pone.0156763 (PMC4892514; doi:10.1371/journal.pone.0156763)
Supplement: S1 Materials and Methods — (PDF) [file pone.0156763.s001.pdf]

Supplementary Materials and Methods

Cell culture

Human umbilical vein endothelial cells (HUVECs) were purchased from Lonza (Clonetics™; Basel, Switzerland) and maintained in EGM-2 BulletKit™ medium (Lonza).

Western blot

Recombinant human histone H3 (Merkmillipore, MW 15508 Da) at 20 ug/ml was preincubated with 100 nM APC at RT for 30 min. The proteins were mixed with 4 x NuPAGE LDS sample buffer (Thermo Fisher Scientific), and boiled for 5 min. They were separated by SDS-PAGE and transferred onto nitrocellulose membranes. After blocking with 5 % skim milk for 1 hour, the blots were incubated with rabbit anti-human H3.3 (1:500, Abcam) at 4℃ for overnight, then incubated with the secondary antibody, goat anti-rabbit IgG conjugated with HRP at RT for 2 hours. Proteins were detected by SuperSignal West Pico Chemiluminescent substrate (Thermo Scientific).
