# Supplementary material for: Histones Induce the Procoagulant Phenotype of Endothelial Cells through Tissue Factor Up-Regulation and Thrombomodulin Down-Regulation
Source: PLoS One. 2016 Jun 3;11(6):e0156763. doi: 10.1371/journal.pone.0156763 (PMC4892514; doi:10.1371/journal.pone.0156763)
Supplement: S1 Fig — (PDF) [file pone.0156763.s002.pdf]

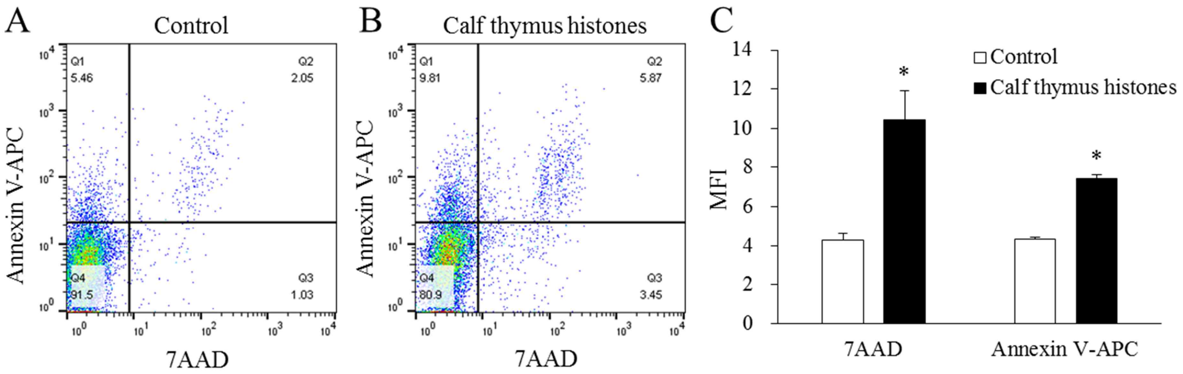

S1 Fig. Histones induced cell damage. EA.hy926 cells were stimulated with or without 50 μg/mL histones for 4 h. Then they were stained with Annexin V and 7AAD, and analyzed by flow cytometry. The dot plots (A: Control, B: calf thymus histones) showed the increase of 7AAD positive and annexin V positive population treated by histone. (C) The expression of 7AAD and annexin V were presented in a bar graph. * $P<0.05$ vs. control.
