# Supplementary material for: Histones Induce the Procoagulant Phenotype of Endothelial Cells through Tissue Factor Up-Regulation and Thrombomodulin Down-Regulation
Source: PLoS One. 2016 Jun 3;11(6):e0156763. doi: 10.1371/journal.pone.0156763 (PMC4892514; doi:10.1371/journal.pone.0156763)
Supplement: S2 Fig — (PDF) [file pone.0156763.s003.pdf]

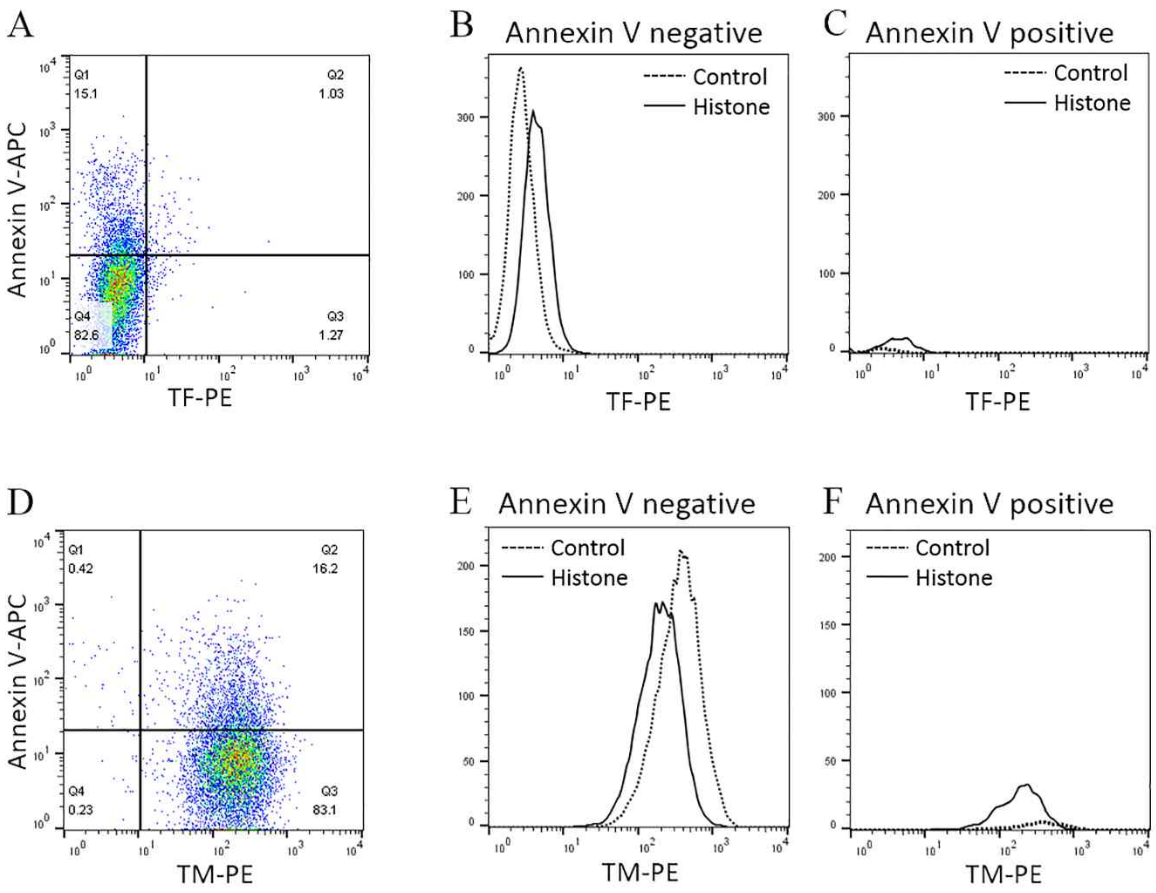

S2 Fig. The expression of TF and TM in undamaged cells by histones-stimulation.

EA.hy926 cells were stimulated with or without 50 μg/mL histones for 4 h. Then they were stained with TF or TM, and Annexin V. (A) The dot plot showed the expression of annexin V with TF of histones-stimulated cells. (B) The histogram showed TF expression of the annexin V negative population gated from A. It was still enhanced by histone stimulation. (C) The histogram showed TF expression of the annexin V positive population gated from A. (D) The dot plot showed the expression of annexin V with TM of histones-stimulated cells. (E) The histogram showed TM expression of the annexin V negative population gated from D. It was still reduced by histone stimulation. (F) The histogram showed TM expression of the annexin V positive population gated from D.
