# Supplementary material for: Histones Induce the Procoagulant Phenotype of Endothelial Cells through Tissue Factor Up-Regulation and Thrombomodulin Down-Regulation
Source: PLoS One. 2016 Jun 3;11(6):e0156763. doi: 10.1371/journal.pone.0156763 (PMC4892514; doi:10.1371/journal.pone.0156763)
Supplement: S3 Fig — (PDF) [file pone.0156763.s004.pdf]

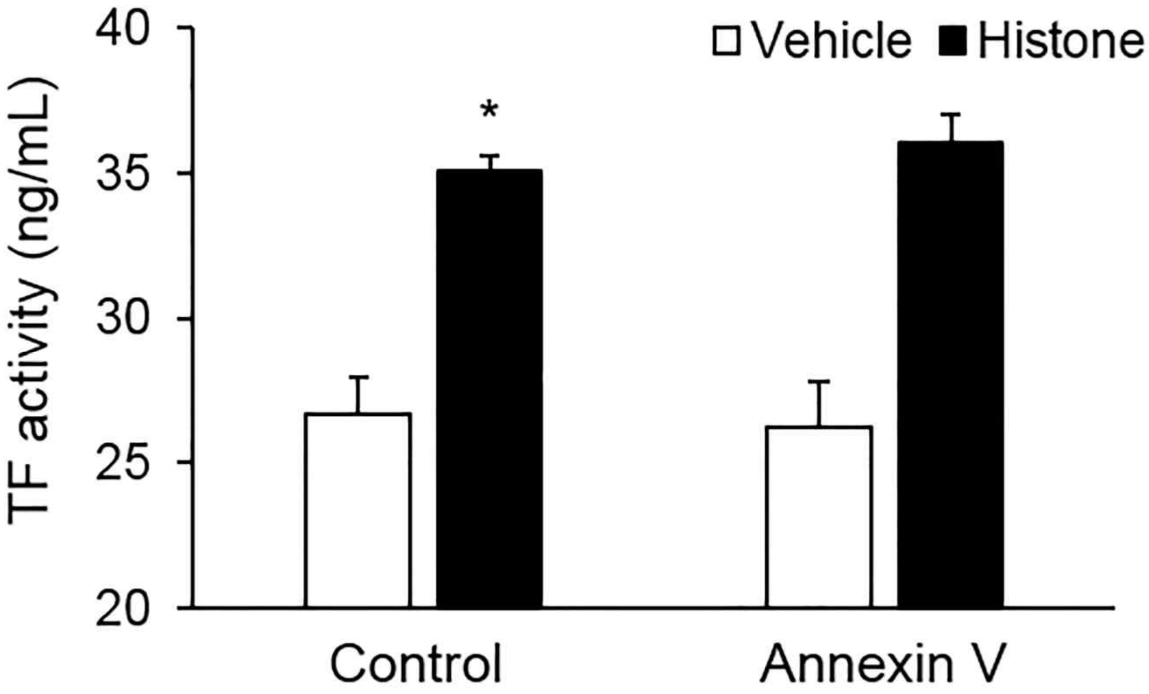

S3 Fig. Annexin V did not affect the induced TF activity of histone-stimulated cells. EA.hy926 cells were stimulated with or without 50 μg/mL histones for 4 h, then incubated with PBS or 10 μg/mL annexin V for 15 min. The TF activity was analyzed using a procoagulant assay. * $P<0.05$ vs. control (histones not treated).
