# Supplementary material for: Histones Induce the Procoagulant Phenotype of Endothelial Cells through Tissue Factor Up-Regulation and Thrombomodulin Down-Regulation
Source: PLoS One. 2016 Jun 3;11(6):e0156763. doi: 10.1371/journal.pone.0156763 (PMC4892514; doi:10.1371/journal.pone.0156763)
Supplement: S4 Fig — (PDF) [file pone.0156763.s005.pdf]

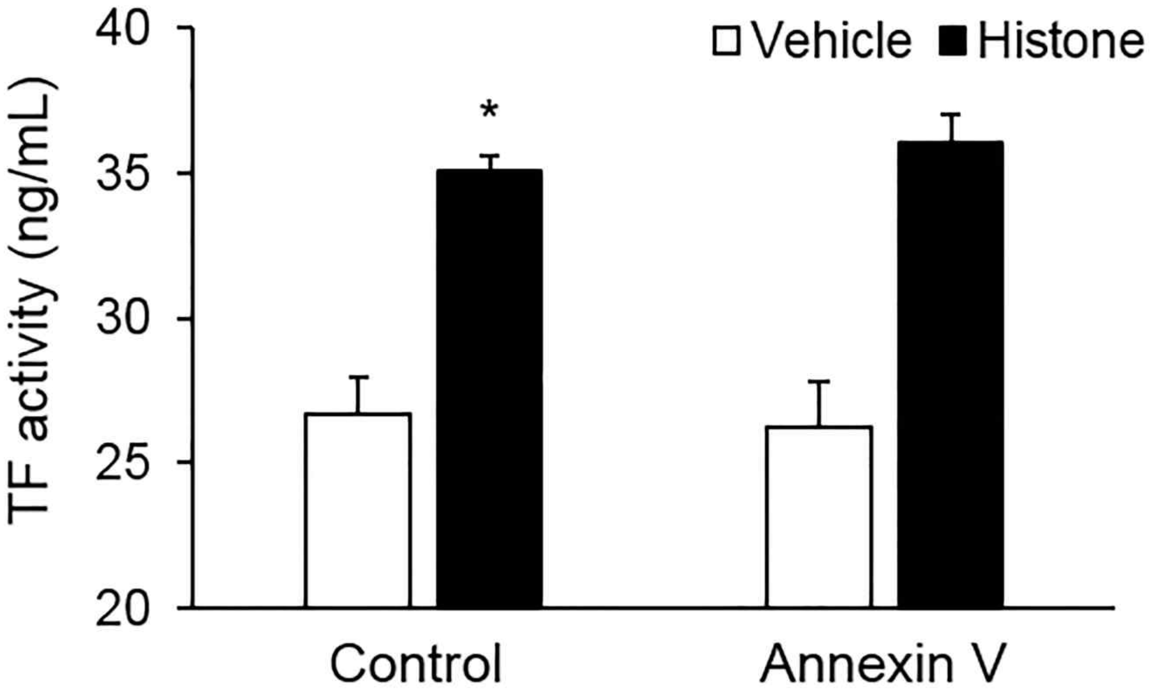

S4 Fig. TF activity of histone-stimulated cells with protein-disulfide isomerase (PDI) inhibitors. EA.hy926 cells were pre-incubated with inhibitors, such as an anti-PDI antibody (RL90, 10 μg/mL), glutathione (7.5 mM), and quercetin (200 μM) for 1 h, prior to stimulation with 50 μg/mL histones for 4 h. The TF activity was analyzed using a procoagulant assay. * P<0.05 vs. control (histones not treated)
