# Supplementary material for: Histones Induce the Procoagulant Phenotype of Endothelial Cells through Tissue Factor Up-Regulation and Thrombomodulin Down-Regulation
Source: PLoS One. 2016 Jun 3;11(6):e0156763. doi: 10.1371/journal.pone.0156763 (PMC4892514; doi:10.1371/journal.pone.0156763)
Supplement: S5 Fig — (PDF) [file pone.0156763.s006.pdf]

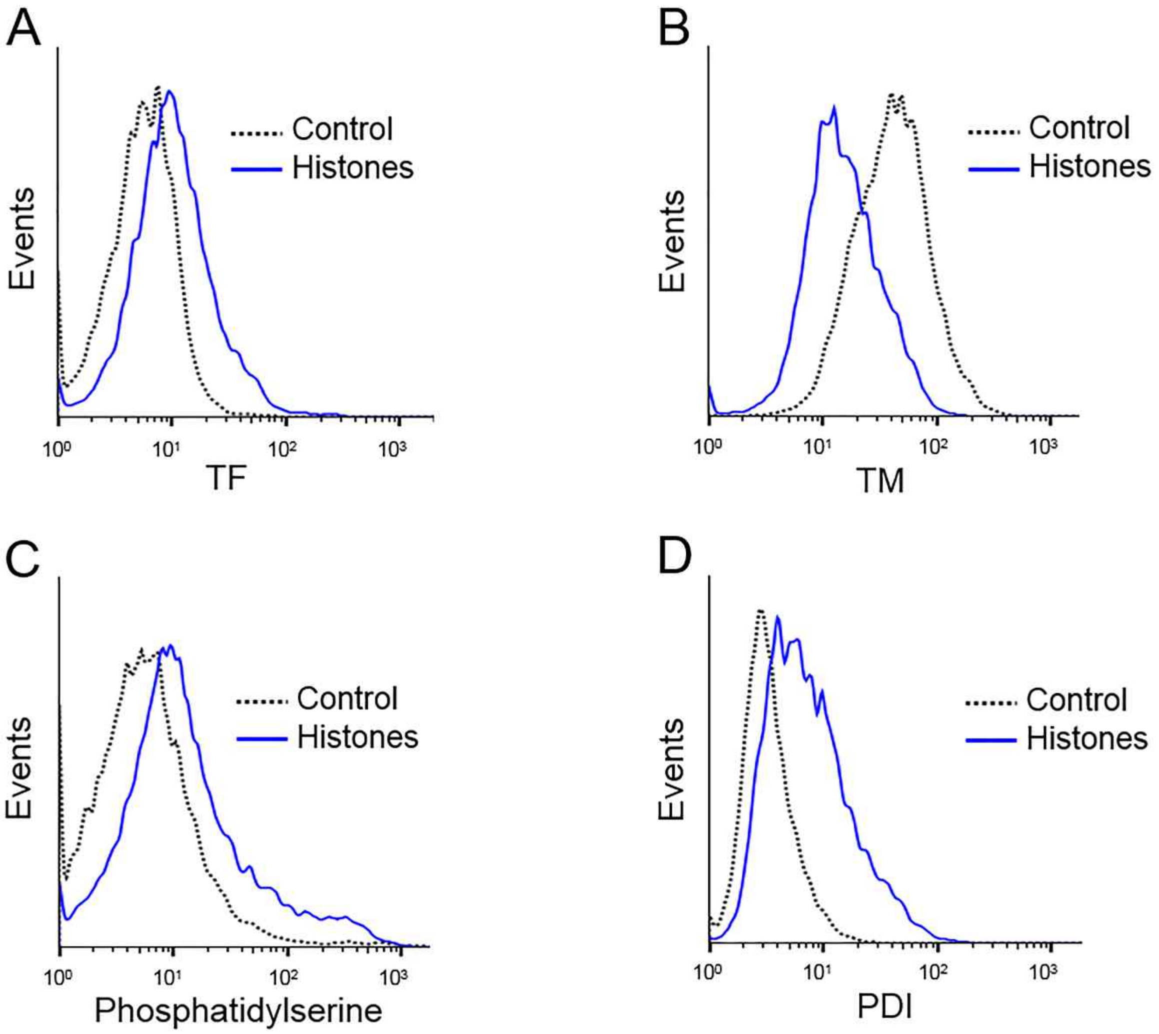

S5 Fig. Histones influenced the expression of several proteins on HUVECs. After HUVECs were stimulated with 50 μg/mL histones for 4 h, surface expression levels of tissue factor (TF; A), thrombomodulin (TM; B), phosphatidylserine (C), and protein-disulfide isomerase (PDI; D) antigen were determined using flow cytometry.
