# Supplementary material for: Histones Induce the Procoagulant Phenotype of Endothelial Cells through Tissue Factor Up-Regulation and Thrombomodulin Down-Regulation
Source: PLoS One. 2016 Jun 3;11(6):e0156763. doi: 10.1371/journal.pone.0156763 (PMC4892514; doi:10.1371/journal.pone.0156763)
Supplement: S6 Fig — (PDF) [file pone.0156763.s007.pdf]

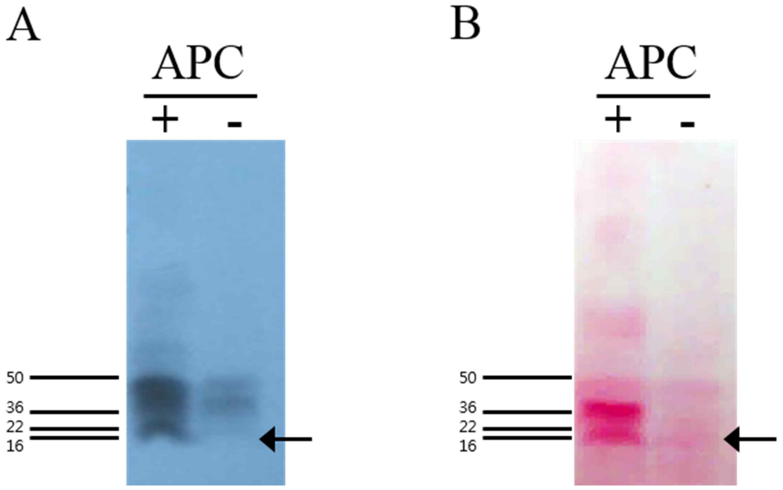

S6 Fig. Western blot analysis for the degradation of histone by activated protein C (APC). The human recombinant histone H3.3 (MW 15.5 KDa) was incubated with or without 100 nM APC at RT for 30 min. (A) Then the histone was visualized by anti-human histone antibody. (B) The membrane of western blot was stained with Ponceau S solution. The arrow indicated intact histone H3. Note that the histone band was weakened under APC treatment.
